# The first two mitochondrial genomes from *Apiotrichum* reveal mitochondrial evolution and different taxonomic assignment of *Trichosporonales*

**DOI:** 10.1186/s43008-023-00112-x

**Published:** 2023-03-31

**Authors:** Qiang Li, Wenqi Xiao, Peng Wu, Ting Zhang, Peng Xiang, Qian Wu, Liang Zou, Mingying Gui

**Affiliations:** 1 Key Laboratory of Coarse Cereal Processing, Ministry of Agriculture and Rural Affairs, School of Food and Biological Engineering, Chengdu University, Chengdu, Sichuan China; 2 Yunnan Plateau Characteristic Agricultural Industry Research Institute, Yunnan Agricultural University, Kunming, Yunnan China; 3 Present Address: School of Food and Biological Engineering, Chengdu University, 2025 # Chengluo Avenue, Chengdu, 610106 Sichuan China

**Keywords:** Mitogenome, Intron, Gene rearrangement, Taxonomy, Phylogenetic analysis

## Abstract

*Apiotrichum* is a diverse anamorphic basidiomycetous yeast genus, and its mitogenome characterization has not been revealed. In this study, we assembled two *Apiotrichum* mitogenomes and compared them with mitogenomes from *Agaricomycotina*, *Pucciniomycotina* and *Ustilaginomycotina*. The mitogenomes of *Apiotrichum gracile* and *A. gamsii* comprised circular DNA molecules, with sizes of 34,648 bp and 38,096 bp, respectively. Intronic regions were found contributed the most to the size expansion of *A. gamsii* mitogenome. Comparative mitogenomic analysis revealed that 6.85–38.89% of nucleotides varied between tRNAs shared by the two *Apiotrichum* mitogenomes. The GC content of all core PCGs in *A. gamsii* was lower than that of *A. gracile*, with an average low value of 4.97%. The *rps3* gene differentiated the most among *Agaricomycotina, Pucciniomycotina* and *Ustilaginomycotina* species, while *nad4L* gene was the most conserved in evolution. The *Ka/Ks* values for *cob* and *rps3* genes were > 1, indicating the two genes may be subjected to positive selection in *Agaricomycotina, Pucciniomycotina* and *Ustilaginomycotina*. Frequent intron loss/gain events and potential intron transfer events have been detected in evolution of *Agaricomycotina, Pucciniomycotina* and *Ustilaginomycotina*. We further detected large-scale gene rearrangements between the 19 mitogenomes from *Agaricomycotina, Pucciniomycotina* and *Ustilaginomycotina*, and fifteen of the 17 mitochondrial genes shared by *Apiotrichum* varied in gene arrangements. Phylogenetic analyses based on maximum likelihood and Bayesian inference methods using a combined mitochondrial gene dataset revealed different taxonomic assignment of two *Apiotrichum* species, wherein *A. gamsii* had a more closely relationship with *Trichosporon asahii*. This study served as the first report on mitogenomes from the genus *Apiotrichum*, which promotes the understanding of evolution, genomics, and phylogeny of *Apiotrichum*.

**Supplementary Information:**

The online version contains supplementary material available at 10.1186/s43008-023-00112-x.

## INTRODUCTION

*Apiotrichum* is an anamorphic basidiomycetous yeast genus, which is widely distributed in nature (Bizarria et al. 2022). Some of the species from the *Apiotrichum* genus are soil-associated species such as *Apiotrichum dulcitum*, *A. laibachii* and *A. loubieri* (Kumla et al. 2020; Takashima et al. 2020), and others are causative agents of human summer-type hypersensitivity pneumonitis (SHP), including *Apiotrichum* *domesticum* and *A. montevideense* (Wu et al. 2023; Almeida et al. 2017; Martinez-Herrera et al. 2021). About 20 species have been described in the *Apiotrichum* genus, and this genus was revised to include new species (James et al. 2016). The genus *Apiotrichum* was revived to accommodate species previously assigned to the brassicae/gracile and porosum clades of the genus *Trichosporon*. The molecular reclassification of the order *Trichosporon*ales placed *Trichosporon* species into three genera: *Cutaneotrichosporon*, *Trichosporon*, and *Apiotrichum* (do Espirito Santo et al. 2020; Li et al. 2020a; Liu et al. 2015). This revival resulted from a major taxonomic revision of the class *Tremellomycetes* based on phylogenetic analyses of a multi-gene dataset, including the internal transcribed spacer region (ITS) rRNA gene, the small subunit (SSU or 18S) rRNA gene, the translation elongation factor 1-α (*TEF1*), the D1/D2 domains of the large subunit (LSU or 26S) rRNA gene, two subunits of RNA polymerase II (*RPB1* and *RPB2*), and cytochrome b (CYTB) gene (Gueho et al. 1992; Peng et al. 2019). Mitochondrial genome (mitogenome) is considered to be a reliable tool for analyzing fungal phylogeny (Abuduaini et al. 2021; Ali et al. 2021). However, so far, the complete mitochondrial genome of *Apiotrichum* species is still unknown.

Mitochondrial genome is called the ‘second genome’ of eukaryotes, which plays an important role in regulating the growth and development, maintaining cell homeostasis and responding to the environment of eukaryotes (Murphy 2009; Ernster and Schatz 1981; McBride et al. 2006; Vowinckel et al. 2021). In addition, the relatively small size of mitochondrial genome, many available molecular markers and parthenogenetic characteristics make mitochondrial genome an important tool for analyzing phylogeny and evolution of fungal species (Cheng et al. 2021; Zhang et al. 2020; Li et al. 2022a). Mitochondrial genome contains abundant genetic information, which have been used to infer the origin and evolution of eukaryotes, including genome size variation, sequence variation, gene arrangement, intron dynamics, tRNA structure, and so on (Wu et al. 2021; Zhang et al. 2021a; Fonseca et al. 2021). Compared with animal mitochondrial genome, fungal mitochondrial genome has more abundant variations in genome size, structure and gene content (Huang et al. 2021). Variable genome sizes, large-scale gene rearrangements, and intron dynamics have been detected between some fungal mitogenomes, even between closely related species (Li et al. 2020b, 2021a). Complex sequence and structural organization make it more difficult to obtain complete fungal mitochondrial genome than animal mitochondrial genome (Wang et al. 2020a). There are more than 30,000 basidiomycetes in nature (Li et al. 2023b; Taylor et al. 2015; Bao et al. 2023). However, so far, less than 150 complete mitochondrial genomes of basidiomycetes have been published in the public database (https://www.ncbi.nlm.nih.gov/genome/browse#!/organelles/). Compared with multiple gene phylogeny, which need multiple polymerase chain reactions (PCR) and pyrosequencings, phylogeny based on mitochondrial genomes is more convenient and practical (James et al. 2006; Li et al. 2022b). The complete mitochondrial genome can be obtained only by second-generation or third-generation sequencing and bioinformatics assembly, which improves the efficiency and accuracy of phylogenetic analysis (Li et al. 2023a). Therefore, mitochondrial genomes have advantages in analyzing fungal phylogenetic relationships and genetic evolution, and more fungal mitochondrial genomes need to be obtained in the future. Up to now, only one complete mitochondrial genome from the *Trichosporonales* order has been published (Yang et al. 2012), which limits our comprehensively understanding of the taxonomic assignment of *Trichosporonales* species.

In the present study, the mitochondrial genomes of two *Apiotrichum* species, including *A. gracile* and *A. gamsii*, were assembled and compared. Comparative mitochondrial genome analysis was conducted to reveal the variations or conservativeness between *Agaricomycotina, Pucciniomycotina* and *Ustilaginomycotina* mitogenomes in genome size, structure, and gene content by comparative mitogenomic analysis. We also reveal the intron dynamics of *Agaricomycotina, Pucciniomycotina* and *Ustilaginomycotina* mitogenomes and analyze the phylogenetic status of *Apiotrichum* in the phylum Basidiomycota based on the combined mitochondrial gene dataset. This study served as the first report on mitogenome from the genus *Apiotrichum*, which will promote the understanding of the evolution, taxonomy and genetics of *Apiotrichum* species and other closely related fugal species from *Agaricomycotina*, *Pucciniomycotina* and *Ustilaginomycotina*.

## MATERIALS AND METHODS

### Mitogenome assembly and annotation

We assembled the compete mitogenomes of *A. gracile* and *A. gamsii* based on the raw sequencing data downloaded from the sequence read archive (SRA) database under the accession numbers of DRR032574 and DRR032575, respectively (Gueho et al. 1992; Middelhoven et al. 2004). The majority of *A. gracile* originated from animal sources, while most strains of *A. gamsii* originated from soil. Unqualified sequences from the raw sequencing data were further removed by using ngsShoRT 2.2 (Chen et al. 2014) and AdapterRemoval v 2 (Schubert et al. 2016), including filtering low-quality sequences and removing adapter reads. Clean reads were obtained after these quality control steps, and downstream analyses were based on the obtained clean reads. NOVOPlasty v4.2.1 (Dierckxsens et al. 2017) was used to assemble the two *Apiotrichum* mitogenomes at the K-mer size of 29. We further obtained the complete mitogenomes of the two *Apiotrichum* species and annotated them according to previously described methods (Li et al. 2018a). Briefly, the protein-coding genes (PCGs), open reading frames (ORFs), tRNAs, rRNAs, and introns of the two *Apiotrichum* mitogenomes were initially annotated using MFannot (Valach et al. 2014) and MITOS (Bernt et al. 2013), both based on the mitochondrial genetic code 4. We then predicted or modified PCGs or ORFs (> 100 aa) based on the NCBI Open Reading Frame Finder (Coordinators 2017). We annotated the functions of PCGs or ORFs by BLASTP searches against the NCBI non-redundant protein sequence database (Bleasby and Wootton 1990). Intron and exon borders of PCGs were determined using exonerate v2.2 (Slater and Birney 2005). We further predicted or verified tRNA genes in the two *Apiotrichum* mitogenomes using tRNAscan-SE v1.3.1 software (Lowe and Chan 2016). Graphical maps of the two *Apiotrichum* mitogenomes were drawn using OGDraw v1.2 (Lohse et al. 2013).

### Sequence analysis

Base compositions of the two *Apiotrichum* mitogenomes and other mitogenomes from *Agaricomycotina, Pucciniomycotina* and *Ustilaginomycotina* were analyzed using DNASTAR Lasergene v7.1 software (http://www.dnastar.com/). We calculated strand asymmetries of these mitogenomes according to following formulas: AT skew = [A − T]/[A + T], and GC skew = [G − C]/[G + C] (Ye et al. 2020). Sequence Manipulation Suite (Stothard 2000) was used to determine codon usages of the two *Apiotrichum* mitogenomes. Core PCGs (*atp6, atp8, atp9, cob*, *cox1, cox2, cox3, nad1, nad2, nad3, nad4, nad4L, nad5, nad6,* and *rps3*) of the 19 mitogenomes from *Agaricomycotina*, *Pucciniomycotina* and *Ustilaginomycotina* were extracted to calculate the nonsynonymous (*Ka*) and synonymous (*Ks*) substitution rates by using DnaSP v6.10.01 (Rozas et al. 2017). We detected genetic distances between each pair of the 15 core PCGs in the 19 mitogenomes using MEGA v6.06 (Caspermeyer 2016), based on the Kimura-2-parameter (K2P) substitution model. Intra-genomic duplications or interspersed repeats of the two *Apiotrichum* mitogenomes were detected by BlastN searches (e-value < 10^−10^) of the two *Apiotrichum* mitogenomes against themselves (Chen et al. 2015). We detected tandem repeats (> 10 bp in length) in the two *Apiotrichum* mitogenomes using Tandem Repeats Finder (Benson 1999) with default parameters.

### Comparative mitogenomic analysis and intron evolution

We compared the 19 previously published *Agaricomycotina, Pucciniomycotina* and *Ustilaginomycotina* mitogenomes to assess variations and conservations between different mitogenomes, including genome sizes, GC contents, base compositions, gene and intron numbers (Additional file 1: Table S1). We further calculated the contribution rate of different regions to the size variation of the two *Apiotrichum* mitogenomes, which is calculated by the following formula: size difference of region/size different of the entire mitogenome *100%. Introns in *cox1* genes of the 19 mitogenomes were classified into different position classes (Pcls) to assess intron evolution and dynamics according to previously described methods (Li et al. 2020b; Ferandon et al. 2010). Briefly, we first aligned the *cox1* genes of the 19 mitogenomes with the *cox1* gene of *Ganoderma calidophilum* (Li et al. 2019a), which was used as the reference gene (Ye et al. 2020), using Clustal W (Thompson et al. 1994). Each Pcl was constituted by introns inserted at the same position of *cox1* reference gene and namely by the insert sites (nt) in the reference gene. The same Pcl was considered as orthologous intron and usually has a high sequence similarity (Ferandon et al. 2010).

### Phylogenetic analysis

In order to investigate the phylogenetic status of *Apiotrichum* species in the phylum Basidiomycota, we constructed a phylogenetic tree of 81 Basidiomycota species from the NCBI database based on the combined mitochondrial gene set (15 core PCGs). The phylogenetic tree was constructed according to previously described methods (Cheng et al. 2021; Qi et al. 2020). *Annulohypoxylon stygium* from the phylum *Ascomycota* was set as the outgroup (Deng et al. 2018). Individual mitochondrial genes (excluding intron regions) were first aligned using MAFFT v7.037 software (Katoh et al. 2019). We further concatenated these aligned mitochondrial genes into a combined mitochondrial dataset using the SequenceMatrix v1.7.8 software (Vaidya et al. 2011). A preliminary partition homogeneity test was conducted to determine potential phylogenetic conflicts between different mitochondrial genes. Best-fit models of partitioning schemes and evolution for the combined mitochondrial dataset were detected using PartitionFinder 2.1.1 (Lanfear et al. 2017). Both the Bayesian inference (BI) and maximum likelihood (ML) methods were used to construct the phylogenetic trees. MrBayes v3.2.6 (Ronquist et al. 2012) was used to conduct the BI analysis. When conducted BI analysis, two independent runs with four chains (three heated and one cold) each were conducted simultaneously for 2 × 10^6^ generations. Each run was sampled every 100 generations. We assumed that stationarity had been reached when the estimated sample size (ESS) was greater than 100, and the potential scale reduction factor (PSRF) approached 1.0. The first 25% samples were discarded as burn-in, and the remaining trees were used to calculate Bayesian posterior probabilities (BPP) in a 50% majority-rule consensus tree. We conducted the ML analysis using RAxML v 8.0.0 (Stamatakis 2014). Bootstrap values (BS) were assessed through an ultrafast bootstrap approach with 1000 replicates.

## RESULTS

### Characterization and PCGs of *Apiotrichum* mitogenomes

**Fig. 1:**
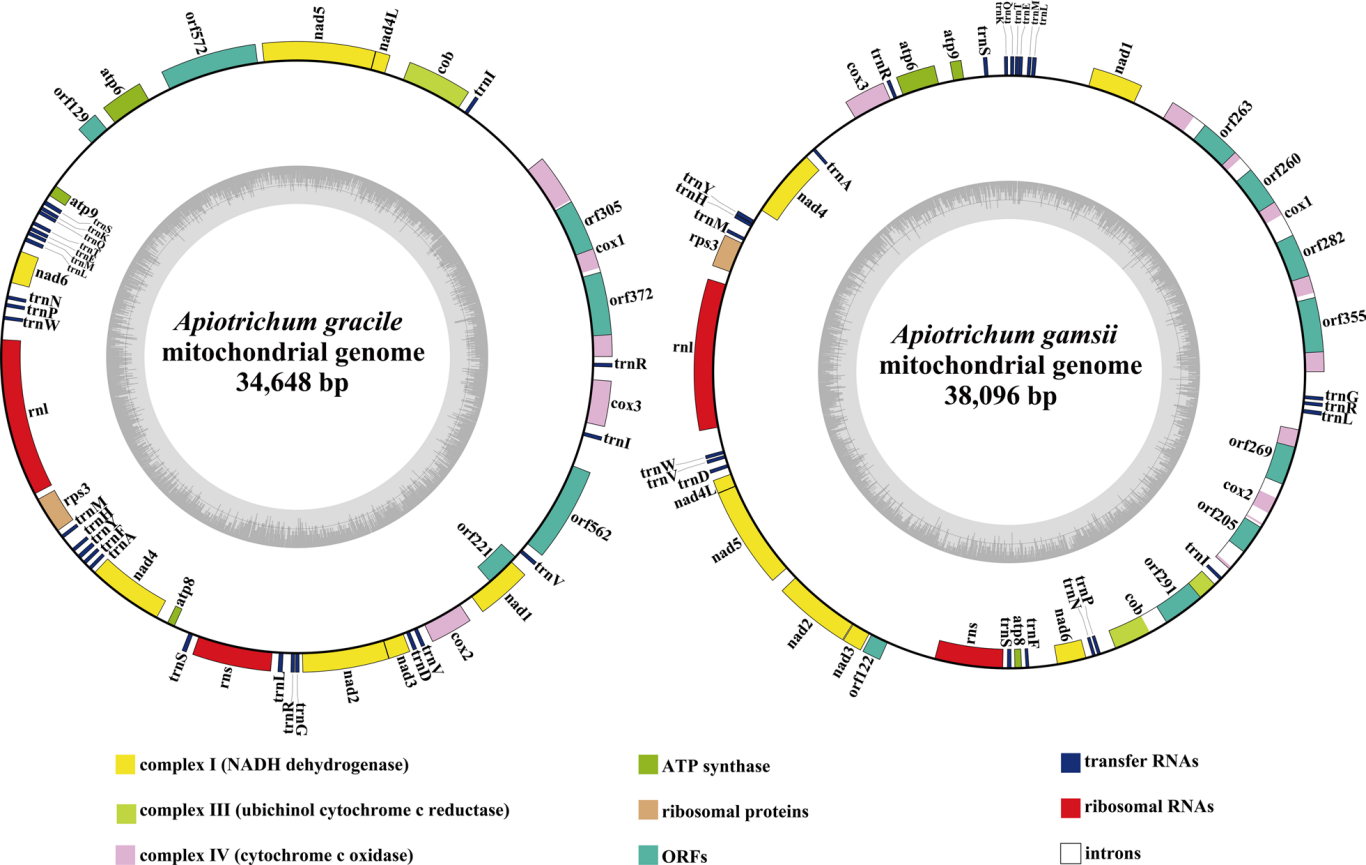
Circular maps of the two *Apiotrichum* mitogenomes. Genes are represented by different colored blocks. Colored blocks outside each ring indicate that the genes are on the direct strand, while colored blocks within the ring indicates that the genes are located on the reverse strand. The inner grayscale bar graph shows the GC content of the mitochondrial sequences. The circle inside the GC content graph marks the 50% threshold

Both the two *Apiotrichum* mitogenomes were circularly organized. The total sizes of the *A. gracile and A. gamsii* mitogenomes were 34,648 bp and 38,096 bp, respectively (Fig. 1). The GC contents of the *A. gracile and A. gamsii* mitogenomes were 31.29% and 27.05%, respectively (Additional file 1: Table S1). Both the AT skew and GC skew of the *A. gracile* mitogenome were negative. The mitogenome of *A. gamsii* contained negative AT skew and positive GC skew in the leading strand. A total of 18 and 16 free stranding PCGs were detected in the mitogenomes of *A. gracile* and *A. gamsii*, respectively (Additional file 1: Table S2). Both the two *Apiotrichum* mitogenomes contained a whole set of core PCGs, including *atp6*, *atp8*, *atp9*, *cob*, *cox1*, *cox2*, *cox3*, *nad1*, *nad2*, *nad3*, *nad4*, *nad4L*, *nad5*, *nad6*, and *rps3*. Nine of the 15 core PCGs had length variations between the two *Apiotrichum* species, of which *rps3* gene had the largest length variation, with 57 bases (Additional file 1: Table S3). The GC content of all core PCGs in *A. gamsii* was lower than that of *A. gracile*, with an average low value of 4.97%. The AT and GC skews of the core PCGs also varied between the two *Apiotrichum* mitogenomes, indicating the two mitogenomes have frequent sequence variations.

The mitogenomes of *A. gracile* and *A. gamsii* contained 3 and 1 non-conserved PCGs encoding proteins with unknown functions, respectively (Additional file 1: Table S2). Two Group I introns were detected in the mitogenome of *A. gracile*, which were located in the *cox1* gene. Two intronic ORFs were detected in the two introns, which encoded LAGLIDADG homing endonucleases. The mitogenome of *A. gamsii* contained 8 introns, 4 of which located in *cox1*, 1 located in *cob*, and the other 3 located in *cox2* gene. Seven out of the 8 introns in the *A. gamsii* mitogenome belonged to the group I. A total of 7 intronic ORFs were detected in the mitogenome of *A. gamsii*, of which 5 encoded LAGLIDADG homing endonucleases and 2 encoded GIY-YIG homing endonucleases.

### RNA genes of the *Apiotrichum* mitogenomes

The two *Apiotrichum* mitogenomes both contained two rRNA genes, namely the large subunit ribosomal RNA (*rnl*), and the small subunit ribosomal RNA (*rns*) (Additional file 1: Table S2). The average lengths of *rns* and *rnl* genes in the two *Apiotrichum* mitogenomes were 1413 bp and 2970 bp, respectively. The *rns* gene of *A. gamsii* mitogenome was 11 bp longer than that of *A. gracile* mitogenome, while the *rnl* of *A. gamsii* mitogenome is 19 bp shorter than that of *A. gracile*.

**Fig. 2:**
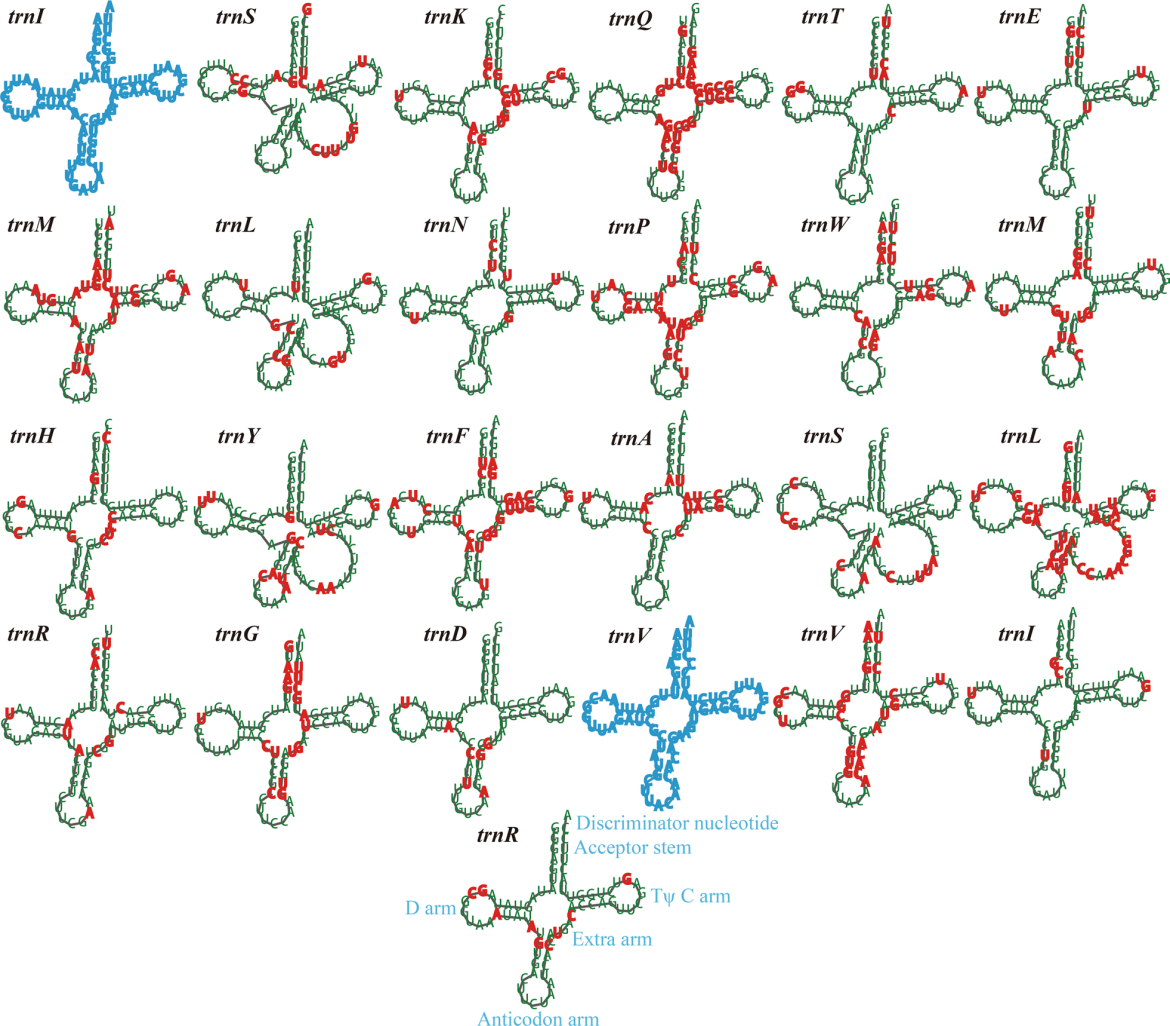
Putative secondary structures of tRNA genes identified in the mitogenomes of two *Apiotrichum* species. The 23 tRNAs in green or red fonts represent tRNAs shared by the two *Apiotrichum* species, while the tRNA in blue font represent tRNA only in *A. gracile*. Residues conserved across the two mitogenomes are shown in green, while variable sites are shown in red. All genes are shown in order of occurrence in the mitochondrial genome of *A. gracile* starting from *trnI*

The mitogenomes of *A. gracile and A. gamsii* contained 25 and 23 tRNA genes, respectively, which were all folded into classical cloverleaf structures (Fig. 2). The two mitogenomes all contained 2 tRNAs with different anticodons coding for arginine, serine and leucine, and contained 2 tRNAs with the same anticodon coding for methionine. In addition, the mitogenome of *A. gracile* contained 2 tRNAs with same anticodons coding for isoleucine and arginine. The size of individual tRNAs ranged from 71 to 88 bp. The *trnS* in the *A. gamsii* mitogenome contained the largest size, which was mainly due to size expansion of extra arms. The 23 tRNA genes shared by the two *Apiotrichum* mitogenomes varied in sites between the two mitogenomes. A total of 345 variable sites were detected in the 23 tRNA genes between the two *Apiotrichum* mitogenomes. Variable sites accounted for 6.85–38.89% of each tRNA shared by the two *Apiotrichum* mitogenomes, and the *trnQ* contained the highest proportion of variable sites between the two *Apiotrichum* mitogenomes (38.89%), followed by the *trnL* (38.82%).

### Overlapping nucleotides and composition of mitogenomes

We detected 2 pairs of overlapping ORFs in the *A. gracile* mitogenome, which were located across the neighboring genes *nad4L*and *nad5* (− 1 bp), as well as between *nad2* and *nad3* (− 1 bp) (Additional file 1: Table S2). One overlapping nucleotide was detected in the *A. gamsii* mitogenome, which was located between *nad4L* and *nad5* (− 1 bp). A total of 9024 bp and 9939 bp of intergenic sequences were detected in the mitogenomes of *A. gracile* and *A. gamsii*, respectively. Intergenic sequences in the two *Apiotrichum* mitogenomes ranged from 3 to 1528 bp. The longest intergenic sequence was located between *cox1* and *trnI* in the *A. gracile* mitogenome.

**Fig. 3:**
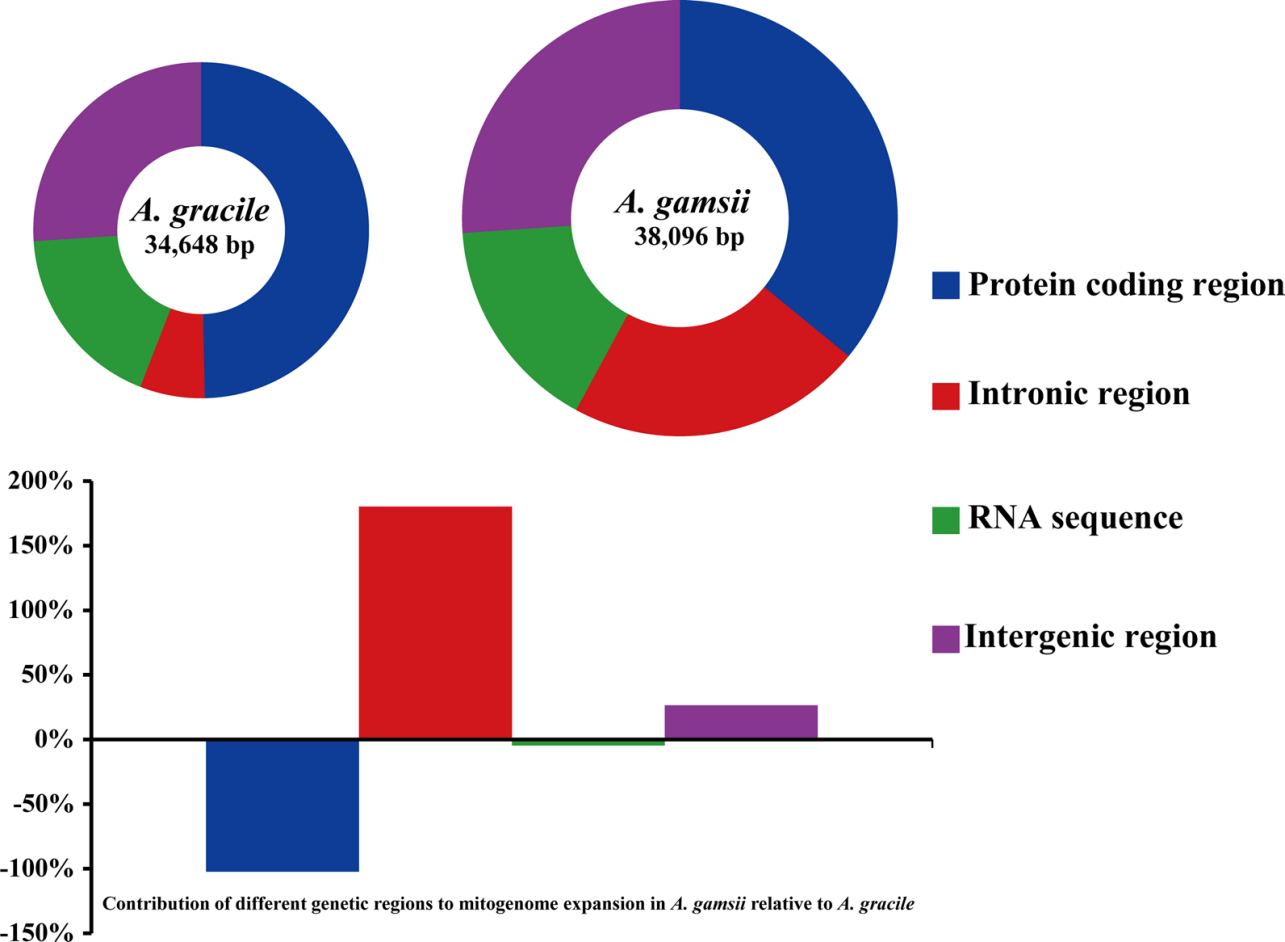
The protein-coding, intronic, intergenic, and RNA gene region proportions of the entire mitogenomes of the two *Apiotrichum* species. The bottom panel shows the contribution of different gene regions to the expansion of the *A. gamsii* mitogenome

The protein coding regions accounted for the largest proportion of *Apiotrichum* mitogenomes, which accounted for 49.66% and 35.90% of the entire *A. gracile* and *A. gamsii* mitogenomes, respectively (Fig. 3). Intergenic regions were the second largest regions in *A. gracile* and *A. gamsii* mitogenomes, which accounted for 26.04% and 26.09% of the entire mitogenomes, respectively. The RNA region was the third largest region in *A. gracile* mitogenome, accounting for 18.05% of the entire mitogenome. While intronic regions accounted for the third largest proportion of the *A. gamsii* mitogenome, occupying 22.00% of the *A. gamsii* mitogenome. The *A. gamsii* mitogenome was 3,448 bp larger than that of *A. gracile*. Intronic regions contributed the most to the size expansion of the *A. gamsii* mitogenome, with the contributing rate of 180.34%. Intergenic regions contributed to 26.54% of the size variation, and protein coding regions contributed to -102.29% of the *A. gamsii* mitogenome expansion.

### Codon usage analysis

ATG was the most frequently used start codon in core PCGs of the 19 species from *Agaricomycotina*, *Pucciniomycotina* and *Ustilaginomycotina* (Additional file 1: Table S4). While the *cox1* gene of *Ustilago bromivora* and *Ustilago maydis* used GTG, the *cox2* and *rps3* genes of *Jaminaea angkorensis* used TTG, and the *nad2* gene of *Ustilago maydis* used GTG as start codons. Most of core PCGs of the 19 species used the TAA as stop codons, followed by the TAG. The start and stop codons varied greatly between different from *Agaricomycotina*, *Pucciniomycotina* and *Ustilaginomycotina* species, even between closely related species. The *nad4* gene of *A. gracile* used TAG as stop codon, while *A. gamsii* used TAA as stop codon. The *cob*, *cox3*, *nad1* and *rps3* genes of *A. gamsii* used TAG as stop codons, while *A. gracile* used TAA as stop codons.

**Fig. 4:**
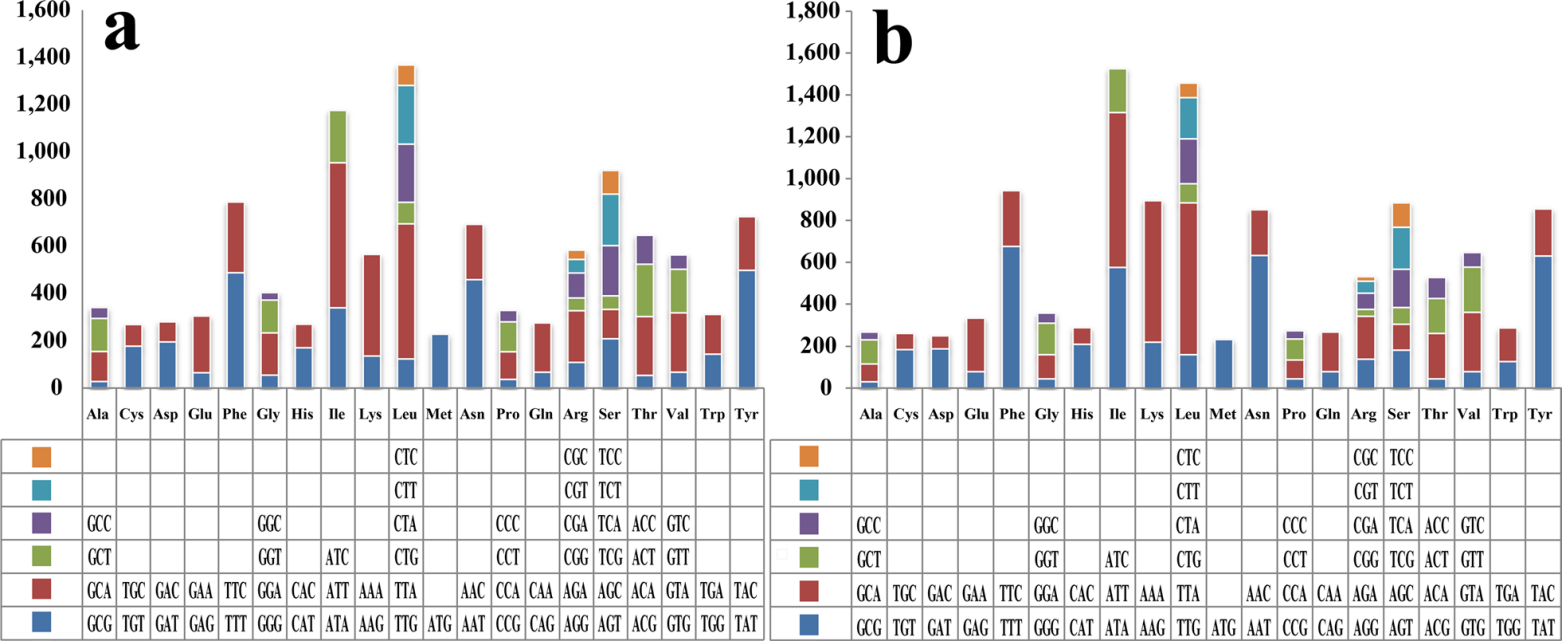
Codon usage in the mitogenomes of two *Apiotrichum* species. Frequency of codon usage is plotted on the y-axis. **a** *Apiotrichum gracile*; **b** *Apiotrichum gamsii*

Codon usage analysis indicated that the most frequently used codons in the two *Apiotrichum* mitogenomes were ATT (for isoleucine; Ile), TTA (for leucine; Leu), TTT (for phenylalanine; Phe), TAT (for tyrosine; Tyr), AAA (for lysine; Lys), and AAT (for asparagine; Asn) (Fig. 4). The frequent use of A and T in codons contributed to a relative high AT content in the two *Apiotrichum* mitogenomes (average: 70.83%).

### Repetitive sequences analysis

Three repeat sequences were detected in each of the two *Apiotrichum* mitogenomes by using BLASTn searches of the two *Apiotrichum* mitogenomes against themselves (Additional file 1: Table S5). The identified repeat sequences ranged from 47 to 246 bp, with pair-wise nucleotide similarities between 87.34 and 96.55%. The largest repeats were located in the intergenic region between *trnP* and *cox2* encompassing the coding region of *trnV*, as well as in the intergenic region between *nad1* and *orf562* encompassing the coding region of *trnV* in the *A. gracile* mitogenome. Repeat sequence accounted for 2.96% and 0.81% of the *A. gracile* and *A. gamsii* mitogenomes, respectively. We detected 7 and 4 tandem repeats in the mitogenomes of *A. gracile* and *A. gamsii*, respectively (Additional file 1: Table S6). The longest tandem repeat sequence was detected in the mitogenome of *A. gracile*, comprising 116 bp, which was located in the intergenic region between *cox1* and *trnI*. Tandem repeat sequences accounted for 1.06% and 0.54% of the *A. gracile* and *A. gamsii* mitogenomes, respectively.

### Genetic distance and evolutionary rates of core genes

**Fig. 5:**
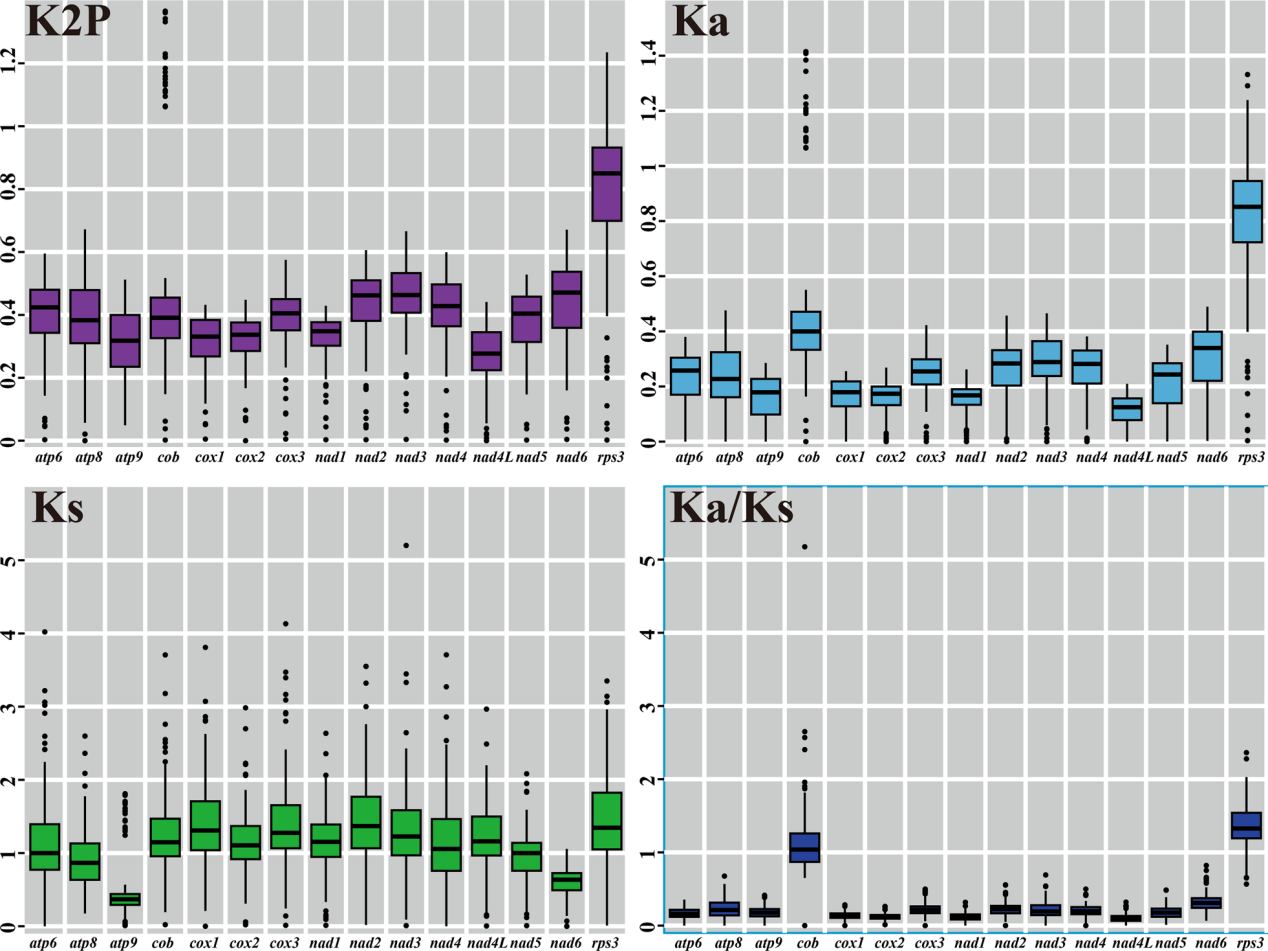
Genetic analysis of 15 protein coding genes conserved in 19 mitogenomes from *Agaricomycotina, Pucciniomycotina* and *Ustilaginomycotina*. K2P, the Kimura-2-parameter distance; *Ka*, the mean number of nonsynonymous substitutions per nonsynonymous site; *Ks*, the mean number of synonymous substitutions per synonymous site

We found the *rps3* gene had the largest average Kimura-2-parameter (K2P) genetic distance among the 15 core PCGs, followed by the *nad6* and *nad3* genes, which showed great differentiation of the 3 core genes among the 19 species from *Agaricomycotina*, *Pucciniomycotina* and *Ustilaginomycotina* (Fig. 5). The *nad4L* gene was found had the smallest average K2P genetic distance among the 15 core PCGs, indicating that the *nad4L* gene was highly conserved between these mitogenomes. The *rps3* gene exhibited the largest non-synonymous substitutions rate (*Ka*) among the 15 core PCGs detected, followed by the *cob* gene. While the *nad4L* gene had the smallest *Ka* value. The synonymous substitution rates (*Ks*) of *nad2* and *rps3* gene were the largest, while that of *atp9* gene was the smallest among the 15 core PCGs detected. We found the *Ka/Ks* values for most of the 15 core PCGs were < 1, including *atp6*, *atp8*, *atp9*, *cox1*, *cox2*, *cox3*, *nad1*, *nad2*, *nad3*, *nad4*, *nad4L*, *nad5*, and *nad6*, which indicated that these genes were subjected to purifying selection. However, the average *Ka/Ks* values for *cob* and *rps3* genes were > 1, indicating the two genes may be subjected to pressure of positive selection.

### Intron dynamics of *cox1* genes

**Fig. 6:**
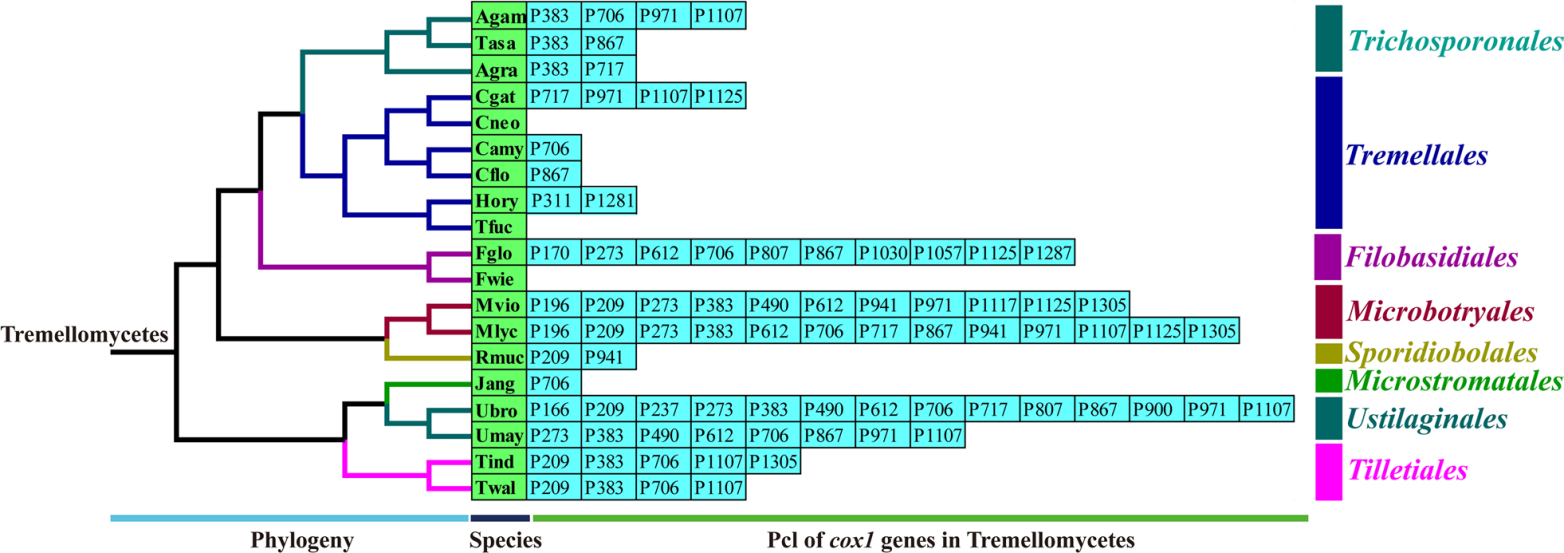
Position class (Pcl) information of *cox1* genes in the 19 species. Introns in *cox1* genes of 19 published mitogenomes were classified into different position classes (Pcls) using the *cox1* gene of *Ganoderma calidophilum* as the reference. Each Pcl was constituted by introns inserted at the same position of corresponding *cox1* gene and named according to its insertion site in the aligned corresponding reference sequence (nt). The Pcls present in more than 1/5 of species were considered as common Pcls, while introns detected in less than 1/5 of species were considered to be rare introns. Phylogenetic positions of the 19 species were established using the Bayesian inference (BI) method and Maximum Likelihood (ML) method based on combined mitochondrial data sets. Species information is shown in Additional file 1: Table S7

A total of 207 introns were detected in the 19 mitogenomes from *Agaricomycotina*, *Pucciniomycotina* and *Ustilaginomycotina*. Several core PCGs were severed as the host genes of introns, which included *atp6*, *atp9*, *cob*, *cox1*, *cox2*, *cox3*, *nad1*, *nad3*, *nad5*, *rns*, and *rnl* genes. The *cox1* gene was found the largest host gene of introns in the 19 mitogenomes. A total of 84 introns were detected in *cox1* genes of the 19 mitogenomes, accounting for 40.58% of the total introns. Intron dynamics in *cox1* gene would have a significantly effect on size or organization variations of mitogenomes from *Agaricomycotina*, *Pucciniomycotina* and *Ustilaginomycotina*. We classified introns of *cox1* genes of the 19 mitogenomes into different position classes (Pcls), which were used to determine whether introns were orthologous (Li et al. 2019a). The 84 introns in *cox1* genes were classified into 25 Pcls (Fig. 6). Different species varied greatly in number or class of Pcls, which indicated that potential intron loss/gain events occurred in evolution of *Agaricomycotina*, *Pucciniomycotina* and *Ustilaginomycotina*.

We considered Pcls present in ≥ 1/5 species as common introns, and Pcls present in < 1/5 species as rare introns in *Agaricomycotina, Pucciniomycotina* and *Ustilaginomycotina* (Fig. 6). A total of 10 common Pcls and 15 rare Pcls were detected in the *cox1* genes of the 19 mitogenomes. The P383 and P706 were the most widely distributed introns in *Agaricomycotina, Pucciniomycotina* and *Ustilaginomycotina*, which were distributed in 9 of the 19 species. However, some rare Pcls could only be detected in one of the 19 species, including P166, P170, P237, P311, P900, P1030, P1057, P1117, P1281, and P1287. We found some rare introns in *Agaricomycotina, Pucciniomycotina* and *Ustilaginomycotina*, including P237, P900, P1030, and P1057, could be detected in distantly related species, such as *Pleurotus eryngii* (Li et al. 2018a; Yang et al. 2016), *Tricholoma matsutake* (Huang et al. 2021), *Armillaria solidipes* (Kolesnikova et al. 2019), and *Ganoderma calidophilum* (Li et al. 2019a) from *Agaricomycotina*, which indicated that potential gene transfer events may occur between distant species in the mitogenome evolution. While some rare introns, such as P166, P170, P311, P1117, P1281, and P1287, could only be detected in mitogenomes from *Agaricomycotina, Pucciniomycotina* and *Ustilaginomycotina*, and no orthologous intron exist in other *Basidiomycota* species. The *cox1* genes of *A. gracile* and *A. gamsii* contained one orthologous intron P383, which may have been obtained from the common ancestor of *Apiotrichum*. However, the mitogenomes of *A. gracile* and *A. gamsii* contained 3 and 1 non homologous intron, respectively, indicating the great differentiation of their intron evolution.

### Gene arrangement and comparative mitogenomic analysis

**Fig. 7:**
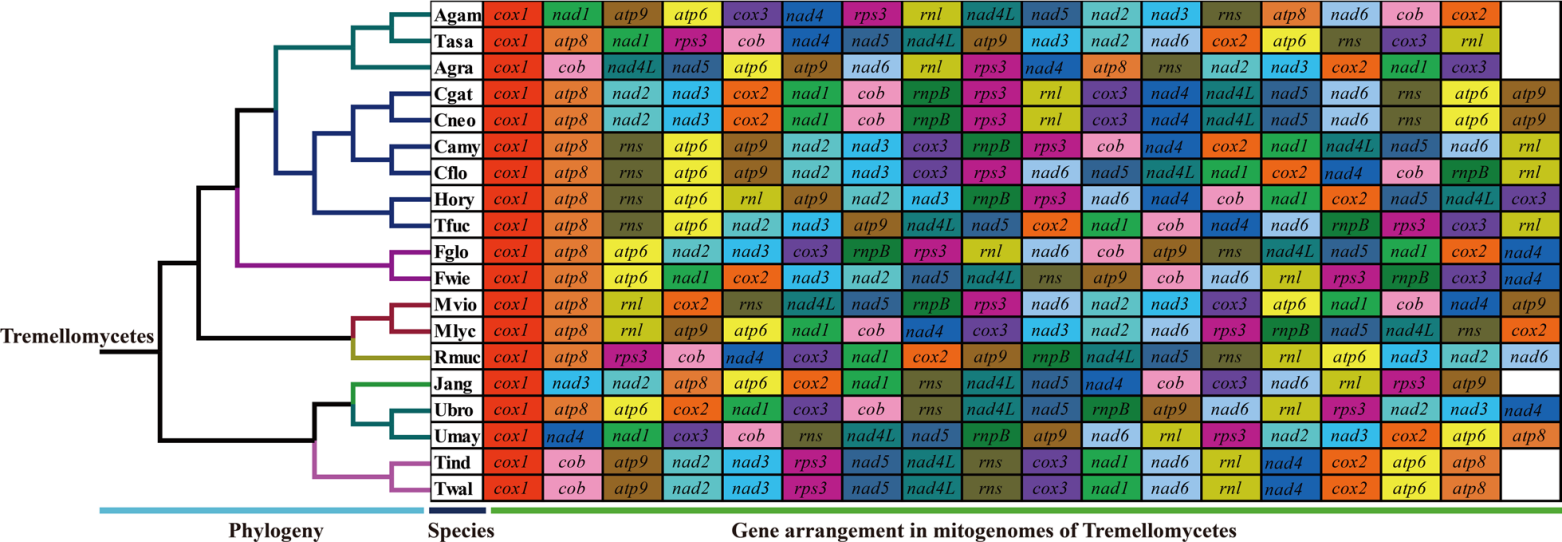
Mitochondrial gene arrangement analyses of the 19 mitogenomes from *Agaricomycotina, Pucciniomycotina* and *Ustilaginomycotina*. The same gene were represented by same color blocks. Phylogenetic positions of the 19 species were established using the Bayesian inference (BI) method and Maximum Likelihood (ML) method based on combined mitochondrial data sets. Species information is shown in Additional file 1: Table S7

We assessed the variation or conservation of mitochondrial gene arrangement in 19 species from *Agaricomycotina*, *Pucciniomycotina* and *Ustilaginomycotina*. Fifteen core PCGs and 2 rRNA genes were included in the mitochondrial gene arrangement analysis. Large-scale gene rearrangements were detected between mitogenomes from different orders, families, or genera (Fig. 7). Species from any different genera had different gene arrangements. In addition, we also observed gene rearrangements between species within the same genera, such as between *Cryptococcus neoformans* and *Cryptococcus amylolentus*, between *Filobasidium wieringae* and *Filobasidium globisporum*, between *Microbotryum cf. violaceum* and *Microbotryum lychnidis dioicae*, as well as between *Ustilago bromivora*and *Ustilago maydis*. Identical gene arrangements were only detected between some closely related species, including between *Cryptococcus gattii* and *Cryptococcus neoformans*, as well as between *Tilletia indica* and *Tilletia walkeri*. We found 15 of the 17 mitochondrial genes varied in gene arrangements between the two *Apiotrichum* species, including gene relocations, position exchanges and so on, indicating that *Apiotrichum* species had undergone large-scale gene rearrangement in the process of evolution.

Comparative mitogenomic analysis indicated that the 19 mitogenomes tested varied greatly in genome sizes, which ranged from 24,874 to 177,540 bp, with an average size of 45,574 bp (Additional file 1: Table S2). The sizes of the two *Apiotrichum* mitogenomes were smaller than the average mitogenome size of the 19 species tested. The GC content of the 19 mitogenomes also varied, ranging from 27.05 to 40.43%, with an average GC content of 33.63%. The two *Apiotrichum* species contained lower GC content than the average GC content of the 19 mitogenomes, and the *A. gamsii* mitogenome contained the lowest GC content among the 19 mitogenomes tested. Eight of the 19 mitogenomes had positive AT skews, while 6 of the 19 mitogenomes had positive GC skews. There were 15–51 PCGs detected in each of the 19 mitogenomes. Introns or intronic ORFs also varied between different mitogenomes. Two rRNA genes and 20–31 tRNA genes were detected in the 19 mitogenomes from *Agaricomycotina*, *Pucciniomycotina* and *Ustilaginomycotina*.

### Phylogenetic analysis

**Fig. 8:**
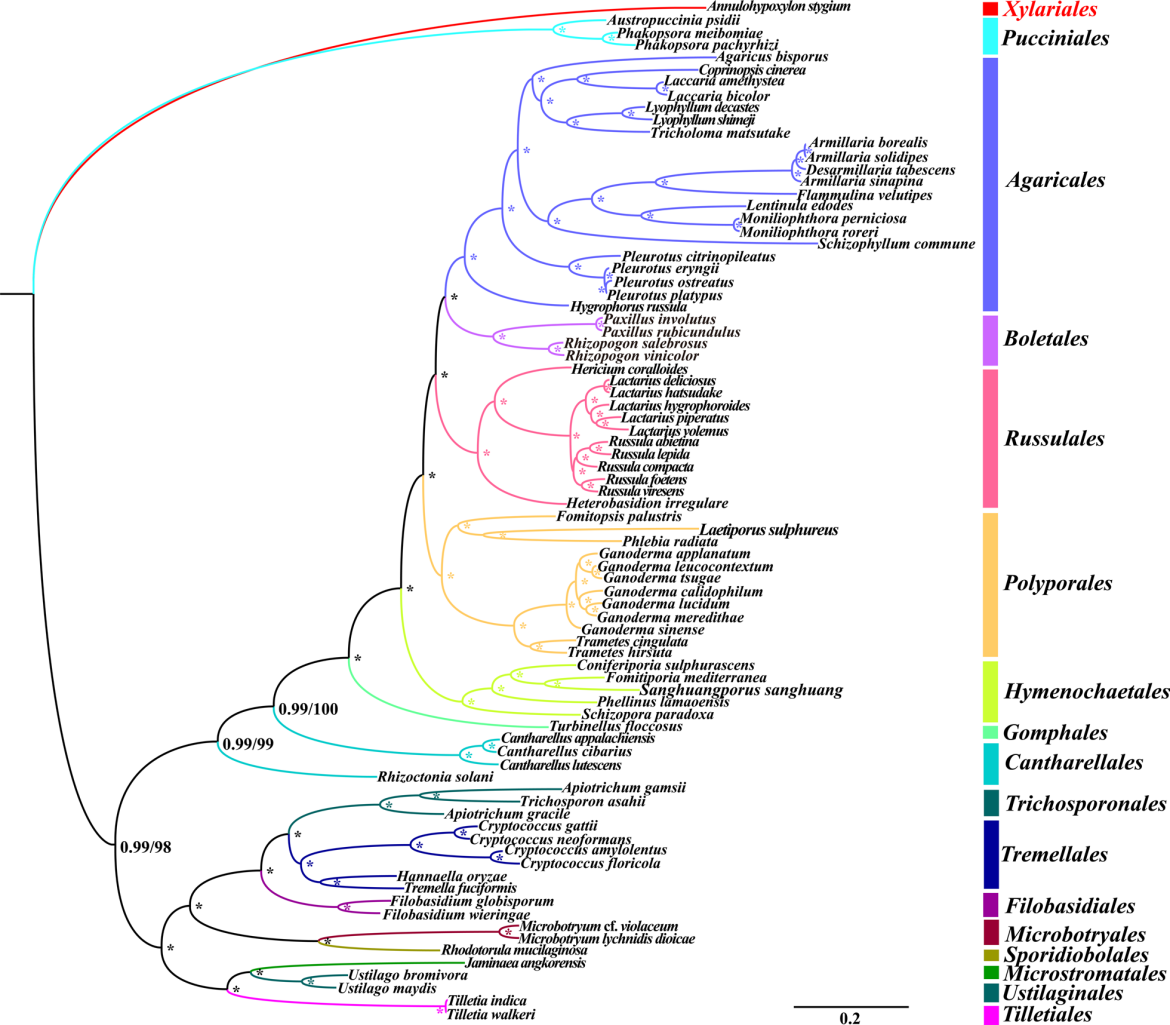
Molecular phylogeny of 81 Basidiomycota species based on Bayesian inference (BI) and Maximum likelihood (ML) analysis of 15 protein coding genes. Support values are Bayesian posterior probabilities (BPP, before slash) and bootstrap values (BS, after slash). An asterisk indicates that the BPP and BS values are 1.00 and 100, respectively. Species and NCBI accession numbers for mitogenomes used in the phylogenetic analysis are provided in Additional file 1: Table S7

We used combined mitochondrial gene datasets (15 core PCGs) to reconstruct phylogenetic tree of 81 Basidiomycota species based on both Bayesian inference (BI) and maximum likelihood (ML) methods, and obtained an identical and well-supported phylogenetic tree (Fig. 8). We found all major clades within the phylogenetic tree had high support rate (BPP ≥ 0.99; BS ≥ 98). The 81 Basidiomycota species in the phylogenetic tree could be divided into 16 major clades, corresponding to the orders *Filobasidiales, Gomphales, Cantharellales, Microbotryales, Sporidiobolales, Pucciniales, Tilletiales, Ustilaginales, Microstromatales, Trichosporonales, Tremellales, Hymenochaetales, Polyporales, Russulales, Boletales*, and *Agaricales* (Additional file 1: Table S7). The two *Apiotrichum* species showed close relationships with species from the order *Tremellales*. According to the phylogenetic tree, *A. gamsii* and *Trichosporon asahii* had a more closer relationship than phylogenetic relationship between the two *Apiotrichum* species. The results showed that the classification of *Apiotrichum* and *Trichosporon* species needed to be further considered.

## DISCUSSION

### Intron dynamics in *Agaricomycotina*, *Pucciniomycotina* and *Ustilaginomycotina* mitogenomes

The mitogenome size of fungi varies greatly, which is considered to be closely related to the accumulation of repeat sequences, gene fragment transfer, dynamic changes of introns and plasmid related genes (Ye et al. 2020; Kolesnikova et al. 2019; Nie et al. 2021). Many fungal introns contain homing endonucleases, which can promote moblity. Up to now, the largest mitogenome among fungi is *Golovinomyces cichoracearum* (332.17 kb) (Zaccaron and Stergiopoulos 2021), which contains 53 introns, followed by *Tuber calosporum* (280.40 kb), containing 58 introns (Li et al. 2020c). Some small mitogenomes in fungi does not contain any introns (Forget et al. 2002). The dynamic changes of introns lead to the huge size variation of fungal mitogenomes. In *Agaricomycotina*, *Pucciniomycotina* and *Ustilaginomycotina*, *Ustilago bromivora* contains the largest mitogenome (177.54 kb) and 29 introns (acc. LT558140 in the NCBI database), while *Cryptococcus neoformans* contains the smallest mitochondrial genome (24.87 kb) and 2 introns (D'Souza et al. 2011), indicating that great size variation and intron dynamics has also occurred in *Agaricomycotina*, *Pucciniomycotina* and *Ustilaginomycotina* species. The size of *Apiotrichum* mitogenomes is smaller than the average size of the 19 mitogenomes tested, indicating that the contraction of mitogenomes have occurred in the evolution of *Apiotrichum* species, which may be due to the loss of introns in *Apiotrichum* species during evolution. Within *Apiotrichum* genus, we also observed the size variations of mitogenomes, in which introns contribute the most to the size expansion of the *A. gamsii* mitogenome relative to *A. gracile* mitogenome. The results showed that intron is an important factor leading to the size variation of fungal mitogenome, which is consistent with the previous research results (Nie et al. 2021). We further assigned different introns into Pcls, and the same Pcl from different species were considered as orthologous introns. Frequent intron loss and gain events were detected among *Agaricomycotina*, *Pucciniomycotina* and *Ustilaginomycotina* species. Further analysis showed that some rare introns in the 19 species could be detected in distant species, indicating potential intron transfer events. In addition, several novel introns have been detected in *Agaricomycotina*, *Pucciniomycotina* and *Ustilaginomycotina* species, which do not exist in other Basidiomycete’s species. The origin and evolution of these novel introns in *Agaricomycotina*, *Pucciniomycotina* and *Ustilaginomycotina* needed to be further analyzed.

### Gene rearrangements of *Agaricomycotina*, *Pucciniomycotina* and *Ustilaginomycotina* mitogenomes

In this study, we found that the *Agaricomycotina*, *Pucciniomycotina* and *Ustilaginomycotina* species tested varied greatly in mitochondrial gene arrangement. Any species from different orders, families or genera had different gene arrangements. In addition, we also observed large-scale gene rearrangements between species within the same genera from the *Filobasidium*, *Cryptococcus*, *Ustilago*, and *Microbotryum*. Fifteen of the 17 mitochondrial genes shared by *Apiotrichum* (15 core PCGs and 2 rRNAs) varied in gene arrangements, including gene relocations, position exchanges and so on, indicating that the mitochondrial gene orders of *Apiotrichum* species were highly variable. Animal mitochondrial gene arrangement is more conservative than fungal mitochondrial gene arrangement, and has been fully studied (Sun et al. 2021; Zhang et al. 2021b, c). Previous studies have proposed several models to reveal mitochondrial gene rearrangement in animals (Xia et al. 2016; Lavrov et al. 2002). However, the rearrangement of fungal mitochondrial genes is relatively complex, and its rearrangement mechanism has not been accurately revealed so far (Wang et al. 2020b; Zhao et al. 2021). Compared with animal mitochondrial genome, fungal mitochondrial genome contains more repetitive sequences, which is considered to be one of the reasons for the large-scale rearrangement of fungal mitochondrial genes (Aguileta et al. 2014). In general, mitochondrial gene arrangement provide important reference to reflect the phylogenetic relationships of fungal species (Li et al. 2018a, b; Wang et al. 2020c).

### Gene variations between *Agaricomycotina*, *Pucciniomycotina* and *Ustilaginomycotina* mitogenomes

The mitogenome of fungi is reported to be obtained from alpha-proteobacteria through endosymbiosis (Munoz-Gomez et al. 2017). Many ancient mitochondrial genes have been reported to have been transferred to the nuclear genome in the process of evolution, which is considered to have a variety of advantages (Adams and Palmer 2003; Adams et al. 2002). However, some protein coding genes, rRNA genes and tRNA genes are still retained in the mitochondrial genome for efficient local control of energy metabolism (Allen 2015). In addition, some gene fragments were observed to have transferred between mitochondrial and nuclear genomes, which promoted the coevolution of the two genomes (Li et al. 2019b). In this study, we did not detect any gene fragments transferring between mitochondrial and nuclear genomes in *Apiotrichum* species. Like most other fungal mitogenomes, the mitogenome of *Apiotrichum* species contains a complete set of core PCGs, which are mainly used for energy metabolism and regulation. These core PCGs varied in sequence composition, codon usage and sequence length. The effects of sequence variations of these core PCGs on protein structure and energy metabolism efficiency need to be further studied. In addition, we also detected possible positive selections on *cob* and *rps3* genes between different *Agaricomycotina*, *Pucciniomycotina* and *Ustilaginomycotina* species, which may be related to the diverse life patterns of species. Species from *Agaricomycotina*, *Pucciniomycotina* and *Ustilaginomycotina* have a variety of life patterns, some are pathogenic, some are saprophytic, some species live in the phyllosphere, some are yeast like, some are mycelial, and some can form basidiocarps (Yurkov and Kurtzman 2019; Millanes et al. 2011). Besides these core PCGs, the two mitogenomes of *Apiotrichum* species varied in rRNA and tRNA sizes and base composition. The size and base composition variations of tRNA have been reported to affect the protein synthesis efficiency (Lin et al. 2021; Liu and Chen 2020), and the effect of tRNA variations on protein synthesis of *Apiotrichum* species needs to be further verified. In addition, we found some non-conserved PCGs in the two *Apiotrichum* mitogenomes, which encoded proteins with unknown functions. The results show that there are still some genes with unknown functions in the mitogenome of *Agaricomycotina*, *Pucciniomycotina* and *Ustilaginomycotina* species that needed to be further resolved, which is very important to understand the origin and evolution of the fungal mitogenome.

### Phylogenetic analysis based on mitochondrial genes

*Apiotrichum* is a diverse fungal genus, widely distributed in nature. Some *Apiotrichum* species are soil-associated species and others are causative agents of human summer-type hypersensitivity pneumonitis (SHP). Accurate identification and classification of *Apiotrichum* species will contribute to the efficient utilization or pathogen control of *Apiotrichum* species (do Espirito Santo et al. 2020). Although *Apiotrichum* species has a variety of life patterns, it has limited or overlapped morphological features for accurate classification only based on morphology. Phylogenetic analyses based on multi-gene dataset placed *Trichosporon* species into three genera: *Cutaneotrichosporon*, *Trichosporon*, and *Apiotrichum* (do Espirito Santo et al. 2020; Li et al. 2020a; Liu et al. 2015). Compared with multi-gene dataset, which requires multiplex PCR amplification and pyrosequencings, the mitogenome of fungi can be obtained only through next-generation sequencing and assembly, which is more convenient and can provide more abundant genetic information than multi-gene dataset. Therefore, phylogenetic analysis based on mitochondrial datasets is a promising choice (Li et al. 2020d, 2021a; b). However, the mitogenome of Basidiomycota is less reported than animal mitogenome or even the *Ascomycetes* mitogenome. Less than 150 complete basidiomycetes mitogenomes have published in the NCBI database, and no *Apiotrichum* mitogenome has been published before. In the present study, we obtained a well-supported phylogenetic tree by using the combined mitochondrial gene dataset (15 core PCGs) based on two phylogenetic inference methods. The mitochondrial gene dataset contained more locus variation information and genetic information than multi-gene dataset. The higher phylogenetic support rate in clades than phylogeny based on multi-gene dataset supported the reliability of phylogenetic relationship inferred from mitochondrial dataset. This phylogeny reconstructed the phylogenetic relationship of basidiomycetes, which showed that *A. gamsii* and *Trichosporon asahii* had a more closer relationship than phylogenetic relationship between the two *Apiotrichum* species, which is inconsistent with the previous studies (Liu et al. 2015), indicating that the classification of *Apiotrichum* species based on mitogenome needs to be considered in the classification of *Trichosporonales* species.

## CONCLUSIONS

In this study, we assembled two *Apiotrichum* mitogenomes and compared them to those from *Agaricomycotina, Pucciniomycotina* and *Ustilaginomycotina*. The two novel mitogenomes displayed considerable size variations, with intronic regions contributing the most to the size expansion of *A. gamsii*'s mitogenome. Furthermore, the base composition of PCGs and tRNAs varied greatly between the two mitogenomes. Core PCGs from *Agaricomycotina, Pucciniomycotina* and *Ustilaginomycotina* showed different rates of evolution, with the *rps3* gene exhibiting the most differentiation and the *nad4L* gene being the most conserved. Additionally, evidence of positive selection was found in the *cob* and *rps3* genes of *Agaricomycotina, Pucciniomycotina* and *Ustilaginomycotina*. Intron loss/gain events and potential intron transfer events were also detected in the evolution of the three phyla. Furthermore, large-scale gene rearrangements were observed between the 19 mitogenomes, and 15 out of the 17 shared mitochondrial genes in *Apiotrichum* had varied arrangements. Finally, phylogenetic analyses based on maximum likelihood and Bayesian inference using a combined mitochondrial gene dataset revealed different taxonomic assignments for the two *Apiotrichum* species, with *A. gamsii* being more closely related to *Trichosporon asahii*. This research was the initial report on mitogenomes from the genus *Apiotrichum*, which advances our knowledge of the evolution, genomics, and phylogeny of *Apiotrichum* and other related taxa.

## Supplementary Information

**Additional file 1.** **Table S1**. Comparison on mitogenomes among 19 species from *Agaricomycotina*, *Pucciniomycotina* and *Ustilaginomycotina*. **Table S2** Annotation and characterization of the two *Apiotrichum* mitogenomes. **Table S3**. Core protein coding gene information of the two *Apiotrichum* species. **Table S4**. Start and stop codons analysis of 19 mitogenomes from *Agaricomycotina*, *Pucciniomycotina* and *Ustilaginomycotina*. **Table S5**. Local BLAST analysis of the *Apiotrichum* mitogenomes against themselves. **Table S6**. Tandem repeats detected in the mitogenomes of *Apiotrichum* using the online program Tandem Repeats Finder. **Table S7**. Species information and GenBank accession number used for phylogenetic analysis in this study.

## Data Availability

The complete mitogenomes of *A. gracile* and *A. gamsii* were deposited in the GenBank database under the accession numbers MZ439918 and MZ439919, respectively.

## Abbreviations

Mitogenome: Mitochondrial genome
PCG: Protein-coding gene
Pcls: Position classes
*Ks*: Synonymous substitution rates
*Ka*: Nonsynonymous substitution rates
BI: Bayesian inference
ML: Maximum likelihood

## Availability of data and materials

All data generated or analyzed during this study are included in this published article [and its supplementary information files].
